# Anthocyanin Color Stabilization by Host-Guest Complexation with *p*-Sulfonatocalix[n]arenes

**DOI:** 10.3390/molecules26175389

**Published:** 2021-09-04

**Authors:** Johan Mendoza, Luis Cruz, Victor de Freitas, Fernando Pina, Nuno Basílio

**Affiliations:** 1 LAQV—REQUIMTE, Departamento de Química, Faculdade de Ciências e Tecnologia, Universidade Nova de Lisboa, 2829-516 Caparica, Portugal; fp@fct.unl.pt; 2 LAQV—REQUIMTE, Departamento de Química e Bioquímica, Faculdade de Ciências, Universidade do Porto, Rua do Campo Alegre, 687, 4169-007 Porto, Portugal; luis.cruz@fc.up.pt (L.C.); vfreitas@fc.up.pt (V.d.F.)

**Keywords:** anthocyanins, host-guest systems, *p*-Sulfonatocalix[n]arenes, color stability

## Abstract

Flavylium-based compounds in their acidic and cationic form bring color to aqueous solutions, while under slightly acidic or neutral conditions they commonly bring discoloration. Selective host-guest complexation between water-soluble *p*-sulfonatocalix[n]arenes (SCn) macrocycles and the flavylium cationic species can increase the stability of the colored form, expanding its domain over the pH scale. The association constants between SCn and the cationic (acid) and neutral basic forms of flavylium-based compounds were determined through UV-Vis host-guest titrations at different pH values. The affinity of the hosts for synthetic chromophore was found to be higher than for a natural anthocyanin (Oenin). The higher affinity of SC4 for the synthetic flavylium was confirmed by ^1^H NMR showing a preferential interaction of the flavylium phenyl ring with the host cavity. In contrast with its synthetic counterpart, the flavylium substitution pattern in the anthocyanin seems to limit the inclusion of the guest in the host’s binding pocket. In this case, the higher affinity was observed for the octamer (SC8) likely due to its larger cavity and higher number of negatively charged sulfonate groups.

## 1. Introduction

**Figure 1:**
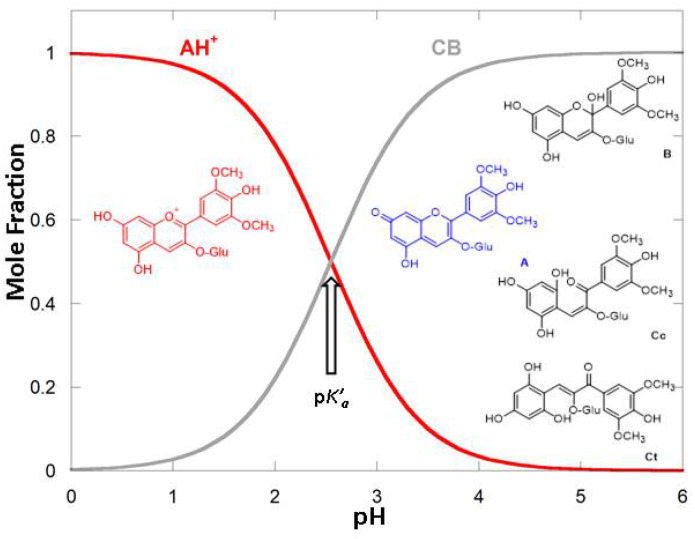
Oenin species distribution along the pH scale at the equilibrium, p*K’_a_* = 2.8.

Anthocyanins belong to the family of flavonoids and comprise the largest group of pigments in the plant kingdom. They are responsible for blue, orange, violet, purple, pink, and red colors in many fruits, leaves, roots, seeds, stems, and flowers [1,2]. However, despite being generally represented in flavylium cation form (AH^+^), anthocyanins are involved in a series of reversible pH-dependent chemical reactions that comprise both the colored flavylium cation and the neutral quinoidal base (A) forms, as well as the colorless hemiketal and *cis*-*trans*-chalcones (B, Cc, and Ct; see Figure 1) [3,4]. The fact that these last colorless species are usually predominant at slightly acidic or neutral pH values strongly hampers more generalized applications of anthocyanins as alternative dyes. The flavylium cation (AH^+^) behaves as a weak acid; in sufficiently acidic mediums it is a unique species, while at higher pH conditions other species are formed (Figure 1) [5,6]. Despite the apparent complexity associated with this multistate system, considering it as a weak acid (AH^+^, with a global acidity constant p*K’_a_*) in equilibrium with a conjugate base (CB, where [CB] = [A] + [B] + [Cc] + [Ct]) greatly simplifies its quantitative analysis. Amongst the CB species only the quinoidal base (A) is colored, but its contribution to CB is minor. In the specific case of the most common anthocyanins, B is often the most abundant species of CB, with A, Cc, and Ct present in significantly lower amounts [7]. Furthermore, because the formation of the *trans*-chalcone (Ct) in anthocyanins is a very slow process, it is possible to define a pseudo-equilibrium between AH^+^ and CB^, with [CB^] = [A] + [B] + [Cc], where no significant amounts of Ct are formed (p*K^^^_a_*) [8]. Nevertheless, when the molar fraction of Ct at the equilibrium is very low p*K^^^_a_* ≈ p*K’_a_*, which is often the case for common anthocyanin [3].

The p*K’_a_* value in anthocyanins is usually found at very low pH and consequently the flavylium domain in the pH scale is short. Amongst the anthocyanins, malvidin-3-O-glucoside (Oenin) is the most abundant pigment in red *Vitis vinifera* grapes and wines [9] and can be obtained from the grape pomace after wine production. 

The use of this, and other anthocyanins, to impart color to food, pharmaceutical, and cosmetic products is limited to highly acidic formulations. For this reason, it is important to increase the flavylium domain along the pH scale. Upward p*K’_a_* shifts in some flavylium derivatives, including anthocyanins, have been previously achieved by complexation with supramolecular receptors such as cucurbiturils [10,11], polymers [12,13] and dendrimers [14].

In this context, calix[n]arenes (Cn) belong to the class of macrocyclic compounds that have shown many interesting recognition properties, serving as host molecules for a wide range of organic and inorganic compounds [14]. Cn macrocycles are typically water-insoluble, therefore their functionalization to improve water solubility is of great interest for biologically relevant applications, such as enzyme inhibition, ion channel blocking, and anti-viral properties, among others [15,16,17,18]. Functionalization with sulfonate, amino, carboxylate, and phosphonate groups was shown to improve their water solubility [19] Among the water-soluble Cn, *p*-sulfonatocalix[n]arenes (SCn) were shown to be particularly soluble (>0.1 M), which together with their low cytotoxic, hemolytic, and non-immunogenicity [20,21,22] has contributed to put these macrocyclic receptors into the focus of supramolecular chemists after their first synthesis by Shinkai and colleagues [23]. Some of these works include important contributions in drug delivery, sensing, and smart material areas, including molecular recognition [24,25,26] and self-assembly [27,28,29,30,31,32,33] phenomena in aqueous solutions at a more fundamental level.

**Scheme 1:**
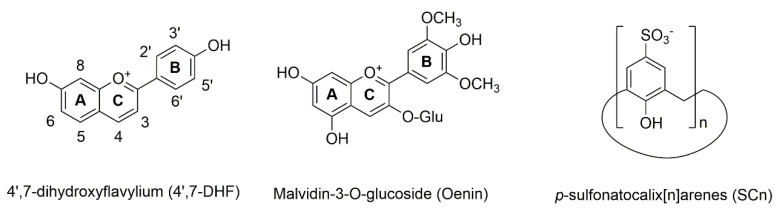
Chemical structures of the flavylium based compounds and the *p*-Sulfonatocalix[n]arenes.

SCn macrocycles are provided with an aromatic binding pocket decorated with SO_3_^−^ groups in the upper rim, being particularly suitable for the binding of positively charged guests that can be stabilized by different interactions such as ion-pairing, cation-π, π-stacking, CH-π, and hydrophobic effects [34,35]. In a previous work, we demonstrated the formation of the photo-responsive host-guest complexes between flavylium-based photo switches and SC4 [36]. On account of the high binding constants observed for selective complexation of synthetic flavylium compounds, in this work we report the results of our studies on the complexation and color stabilization of a synthetic flavylium cation and a natural anthocyanin with SC4, SC6, and SC8 (Scheme 1).

## 2. Material and Methods

The flavylium compound 4′,7-dihydroxyflavylium (4′7-DHF) was synthesized as described elsewhere [37]. Malvidin-3-O-glucoside (Oenin) was obtained after extraction and purification from young wine (*Vitis vinifera L. Cv.* Touriga Nacional) using semipreparative chromatography in a reverse-phase C18 column (250 mm × 4.6 mm i.d.), as previously published [38].

The *p*-sulfonated calixarenes (SCn) macrocycles were obtained by ipso-sulfonation of their respective *p*-tert butylcalix[n]arenes in sulfuric acid, as previously described [39,40,41]. Briefly, 15 mL of H_2_SO_4_ were mixed with 0.5 g of the respective *p*-tert butylcalix[n]arene. The reaction mixture was stirred for 6–18 h at 70–80 °C. The reaction was monitored by taking an aliquot of reaction and placing it in water; it was complete when no water-insoluble material was detected. The reaction was added to 100 mL of water, and the SCnNa salt was obtained by neutralizing the mixture with Na_2_CO_3_ and further filtration. The powder was redissolved in water and treated with activated charcoal for its discoloration; the solution was filtered through Celite. The sodium salts were purified by recrystallization from water/methanol mixtures.

SC4. Following the general procedure, this calixarene was obtained in 66% yield. ^1^H NMR (400 MHz, D2O) δ (ppm): 3.9 (s, 8H, ArCH2Ar), 7.45 (s, 8H, ArH).

SC6. Following the general procedure, this calixarene was obtained in 59% yield. ^1^H NMR (400 MHz, D2O) δ (ppm): 3.88 (s, 12H, ArCH2Ar), 7.41 (s, 12H, ArH).

SC8. Following the general procedure, this calixarene was obtained in 63% yield. ^1^H NMR (400 MHz, D2O) δ (ppm): 4.11 (s, 16H, ArCH2Ar), 7.64 (s, 16H, ArH).

Stock solutions of Oenin and 4′7-DHF were prepared in 0.1 M HCl using Millipore water. The solution’s pH was adjusted by the addition of HCl, NaOH, or citrate buffer (0.01 M) to ensure pH values between 1 and 6. All pH changes were measured by a Radiometer Copenhagen PHM240 pH/ion meter (Brønshøj, Denmark). The UV-Vis spectra were recorded on an Agilent Varian-Cary 100 spectrophotometer (Palo Alto, CA, USA). ^1^H NMR spectra were obtained using a Bruker AMX 400 (Billerica, MA, USA) instrument operating at 400.13 MHz. All the sample solutions were prepared in deuterated water and pD was set to pD = 1 with 0.1 M of DCl.

## 3. Results and Discussion

**Figure 2:**
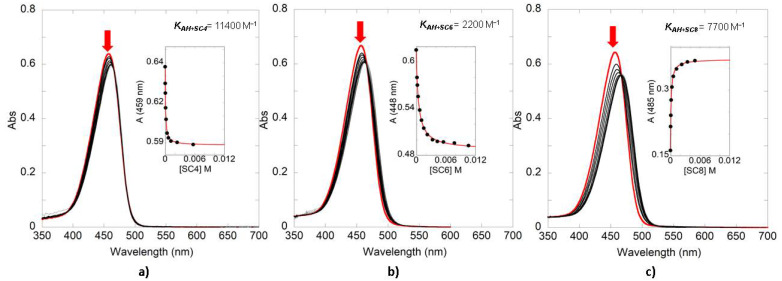
UV-Vis spectral variations of a 4′7-DHF (1.2 × 10^−5^ M) solution at pH=1 upon addition of increasing concentrations of (**a**) SC4, (**b**) SC6 and (**c**) SC8. The inset shows the fitting for the calculation of the association constant considering a 1:1 H-G interaction.

The host-guest interaction between a macrocycle as the SCn and a chromophore may result in UV-Vis spectral variations that can be used to conveniently determine the association constants (*K*) through host-guest titrations monitored by this spectroscopic technique. The interaction between SCn and the flavylium cation form of the model compound 4′7-DHF and the anthocyanin Oenin was investigated at pH 1, where flavylium was the only species in the flavylium multi-state [3]. Figure 2 shows the variation in the absorbance spectra after titration of 4′7-DHF solutions with increasing amounts of SC4, SC6, and SC8. The absorption spectra in all the cases show bathochromic and hypochromic effects after the gradual addition of the host.

The insets in Figure 2 plot the variation of the absorbance at a single wavelength against the concentration of macrocycle. These data can be fitted using a host-guest binding model to obtain the respective association constants ($K_{AH^{+}SCn}$) of SC4, SC6, and SC8 with the guest 4′7-DHF. Equation (1), reported by Oliveira and colleagues [42] for the co-pigmentation effect, can be used to fit the UV-Vis data obtained after titration with the hosts (see Electronic Supplementary Information ESI for details).

(1) $$A_{\lambda }=A_{0}\frac{\left(1+r_{AH^{+}SCn}K_{AH^{+}SCn}\left[\mathrm{SCn}\right]\right)}{\left(1+K_{AH^{+}SCn}\left[\mathrm{SCn}\right]\right)}$$

where *A_λ_* is the absorbance at a specific wavelength (the one to be fitted), $A_{0}$ is the absorbance of the flavylium dye when [SCn]=0, $r_{AH^{+}SCn}$ is the ratio of the molar absorptivity coefficient (ε) of the AH^+^ alone and the AH^+^SCn complex, $K_{AH^{+}SCn}$ is the association constant of the flavylium cation with the macrocycle, and [SCn] is the equilibrium concentration of SCn.

**Figure 3:**
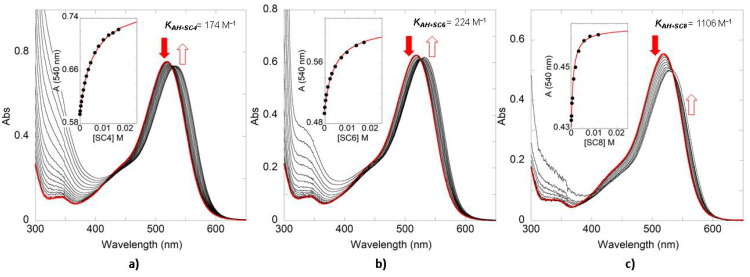
UV-Vis spectral variations of an Oenin solution at pH = 1 upon addition of increasing concentrations of (**a**) SC4 (Oenin = 2.2 × 10^−5^ M), (**b**) SC6 (Oenin = 1.8 × 10^−5^ M) and (**c**) SC8 (Oenin = 1.6 × 10^−5^ M). The inset shows the fitting for the calculation of the association constant with a 1:1 H-G model.

The same procedure was applied for the determination of the association constants with the anthocyanin Oenin. The UV-Vis absorption spectra obtained after titration with the different SCn macrocycles are presented in Figure 3. The association constants obtained from both Figure 2 and Figure 3 are summarized in Table 1. While for the 4′7-DHF model compound, the higher association constant is observed for SC4, Oenin association constant with the SCn macrocycles increases with the number of phenolic units in the host. The observed association constants to bind the synthetic flavylium guest were found to be higher than the ones observed for the anthocyanin, with the larger selectivity for the synthetic dye being observed for SC4 (65-fold) and the lower for SC8 (7-fold). Notwithstanding the moderate selectivity observed for the different SCn homologues, it is interesting to note that, in the case of DHF, the higher association constant was obtained for the complex with SC4 (1.1 × 10^4^ M^−1^). This particular result is in good agreement with the association constants previously reported for similar guests [36]. The small cavity and the proximity of the sulfonate groups in the host as well as the small size of the guest can be responsible for the higher binding constant. On the other hand, flexibility and overall charge may also play a role in the slightly favorable affinity of 4′7-DHF towards SC8 when compared with SC6. In the case of Oenin, the cavity size of the SCn and/or the number of sulfonate groups seems to be the determining factor controlling the stability of the complexes formed with the Oenin, with the larger SC8 showing the higher affinity for the anthocyanin.

**Figure 4:**
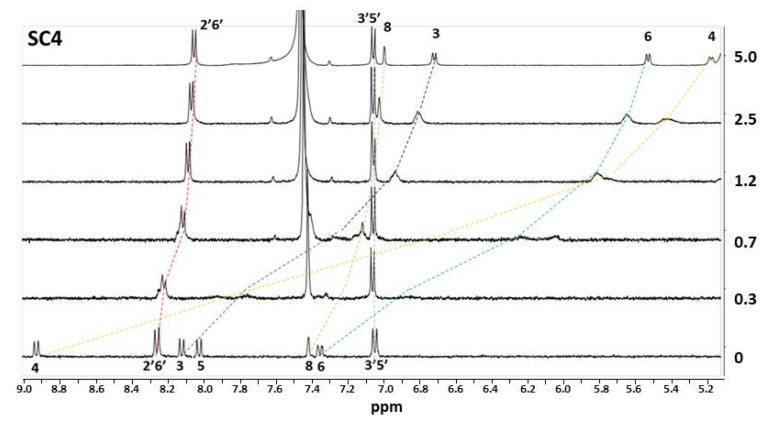
^1^H NMR spectra variations of 4′7-DHF (6 × 10^−4^ M) solution upon titration with increasing concentrations of the host: 0, 0.3, 0.7, 1.2, 2.5, and 5 equivalents of SC4.

^1^H NMR spectroscopy is a powerful tool to identify the binding sites in the structure of the SCn and phenolic compounds complexes, analyzing the complexation-induced changes in the chemical shifts (Δδ) of the guest protons [35,36,39]. The protons with the largest Δδ are mostly affected by the ring current effect of the aromatic nuclei in the SCn macrocycles. In this sense, ^1^H NMR experiments for the complexes 4′7-DHF-SCn were conducted at pD = 1 in D_2_O. (Figure 4) shows the ^1^H NMR spectra of a solution with a fixed concentration of 4′7-DHF and increasing concentrations of the host SC4. The guest molecule experiences a significant complexation-induced shift owing to the ring current effect of the aromatic nuclei of the SC4, indicating the encapsulation of the 4′7-DHF guest into the host cavity. The most affected ^1^H NMR signals, after complexation with the host molecule, are the H3, H4, H5, and H6, located around the A and C rings in the flavylium backbone. The H4 and H6 signals seem to be the more affected than the protons around the B ring. The fast/medium exchange dynamics between the free and complexed guest produce a broadening of some proton signals in the ^1^H NMR spectra, as frequently observed for the association of guest molecules with sulfonated calixarenes [43,44].

Similar results were observed for the complexation of the 4′7-DHF with SC6 and SC8 (ESI). The most remarkable difference between the complexation with SC4 and SC6 is that that the H2′6′ protons signal is most affected in the latter, in addition, in the SC8 complexation the H3′5′ is affected by the inclusion in the larger cavity. These results seem to indicate less specific binding geometries between the guest and the larger macrocycles. 

**Figure 5:**
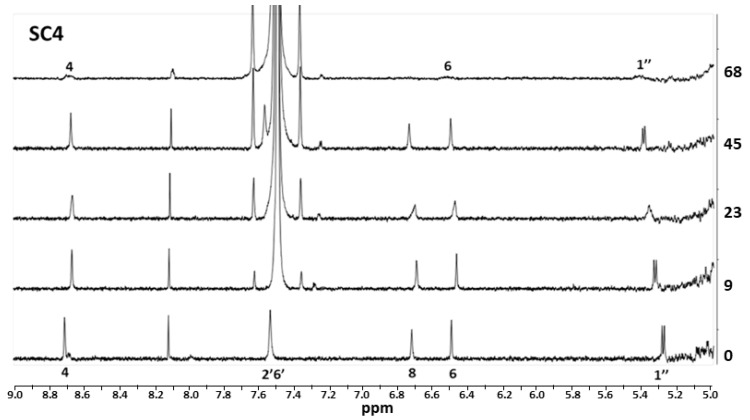
^1^H NMR spectra variations of Oenin (2.2 × 10^−4^ M) solution upon titration with increasing concentrations of the host: 0, 9, 23, 45, and 68 equivalents of SC4.

For the complexation of the SCn with the anthocyanin, no high ∆δ values were observed upon titration, even for higher concentrations of the host (see Figure 5). These results relate to two different hypotheses, (i) the aggregation effect observed in anthocyanins at high concentrations is due to the interaction with the host [45], or (ii) the interaction is not necessarily an inclusion of the guest in the cavity, but rather an ion-pair exclusion complex formed between the dye and the negatively charged sulfonate groups of the SCn. 

**Figure 6:**
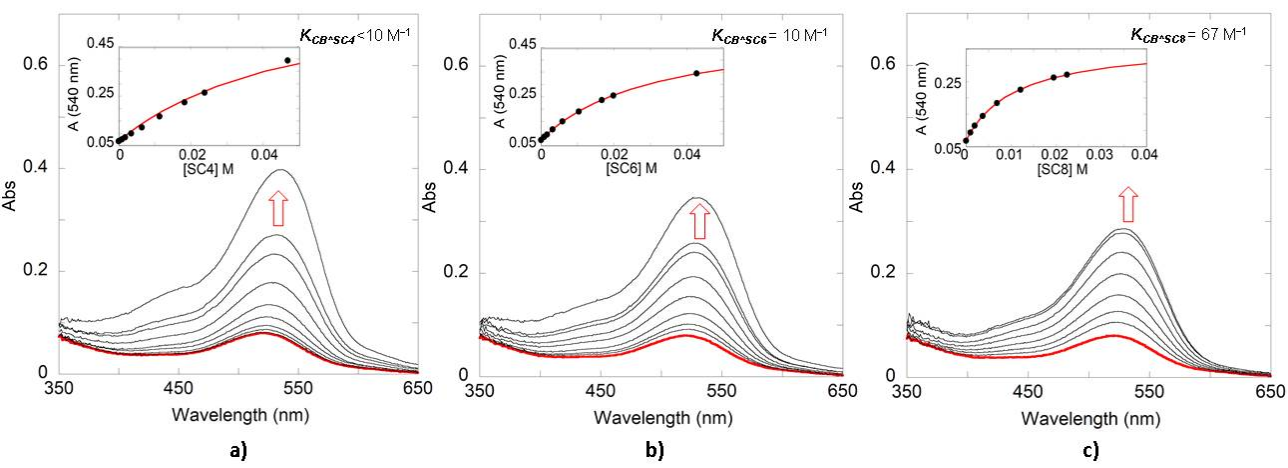
UV-Vis spectral variations of a Oenin upon addition of increasing concentrations of the SCn macrocycles. (**a**) Oenin 2 × 10^−5^ M vs. SC4, pH = 3.52, (**b**) Oenin 1.8 × 10^−5^ M vs. SC6, pH = 3.43 and (**c**) Oenin 1.8 × 10^−5^ M vs. SC8, pH = 3.78. The inset shows the fitting for the calculation of the association constant considering a 1:1 H-G interaction using Equation (2).

The pH-dependent stability of the Oenin flavylium cation was investigated at pH values above pH = 1. In the case of the anthocyanins, the flavylium cation reaches the pseudo-equilibrium in a few seconds or hours depending on the final pH, producing the conjugate base species (CB^) [45,46]. Due to the binding properties of the SCn to selectively recognize cationic organic molecules, the flavylium species can be expected to form host-guest complexes with higher affinity than the CB^ species. The calculation of the apparent association constant of the CB^ species with the macrocycles can be determined from host-guest titrations at slightly acidic pH values. Titration of pseudo-equilibrated solutions at pH ≈ 3.5 were performed, where the molar fraction of the Oenin AH^+^ was around 10% [7]. Figure 6 shows the UV-vis spectra obtained after titration with SC4, SC6, and SC8. As can be observed, the visible absorption band assigned to the flavylium cation increases as the concentration of SCn is augmented because of the higher stability of these complexes. The insets show the plot of absorbance, at a specific wavelength, plotted against the concentration of macrocycle and the respective data fitting using Equation (2) [42]:

(2) Aλ=A0(1+rAH+SCnKAH+SCn[SCn])[H+]+rCB^+rCB^SCn[SCn](1+KAH+SCn[SCn])[H+]+(1+KCB^SCn[SCn]) Ka^

where rCB^ and rCB^CP are the ratio of the ε of the free and complexed CB^^^ species and the ε of AH^+^SCn, respectively, while KCB^SCn is the association constant of the macrocycles with the CB^ species and the Ka^ is the acid ionization constant at the pseudo-equilibrium.

The results obtained for Oenin, from the data reported in Figure 6, are presented in Table 2. As expected, the KCB^SCn were lower that the $K_{AH^{+}SCn}$ presented in Table 1. As observed for the complexation of the *SCn* macrocycles with the flavylium cation, the Oenin CB^ association constants also show that the basic species display higher affinity for the larger SC8 homologue, providing evidence of the higher ability of this receptor to bind the different Oenin species. 

**Figure 7:**
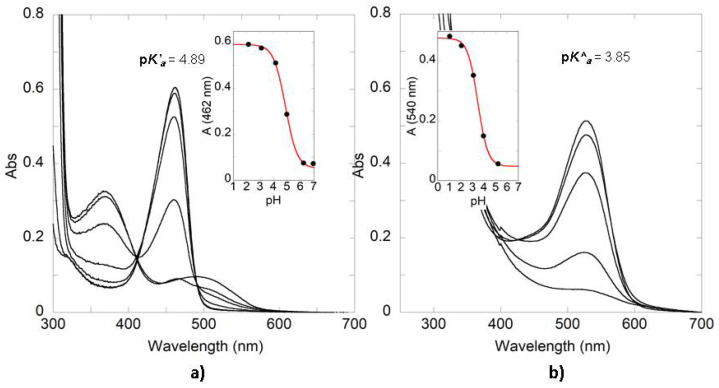
UV-Vis spectral variations of equilibrated and pseudo-equilibrated solutions obtained after direct pH jumps from pH=1 to higher pH values (citrate buffer 0.01M). (**a**) 4′7-DHF (1.15 × 10^−5^ M) in presence of SC4 46 mM and (**b**) Oenin (1.7 × 10^−5^ M) in presence of SC8 6.5 mM. The insets show the fitting for the calculation of p*K’_a_* or p*K^^^_a_*.

The previously obtained results were confirmed by studying the effect of the SCn in the stabilization of the flavylium cation along the pH scale. The UV-Vis spectral variations of 4′7-DHF and Oenin in the presence of the SCn with the highest *K_AH+SCn_* are presented in Figure 7 (remaining results obtained for the other SCn are included in the ESI). As can be observed, the p*K’_a_* or p*K^^^_a_* significantly increased in the presence of the SCn as a result of the higher affinity of these receptors for the positively charged flavylium species. The host-guest complexation of the 4′7-DHF model compound with the SCn exhibits a p*K’_a_* shift of up to 1.8 units (considering the p*K’_a_* = 3.05 [47] for the free dye). This is in accordance with the association constants, where the higher p*K’_a_* shift is observed for those receptors with higher affinity and selectivity for AH^+^. Nevertheless, in Oenin, the p*K^^^_a_* shifts after the complexation is smaller, reaching 1.25 units up in the case of SC8 (based on the value of p*K^^^_a_* = 2.6 [7] for oenin), because of the association constants for the anthocyanin are lower than those observed for the synthetic flavylium. Nevertheless, the higher p*K^^^_a_* shift observed in the presence of the larger SC8 host seems to indicate this particular SCn as an interesting scaffold for further investigation in the context of anthocyanin binding and color stabilization. The UV-vis spectrum relative to the higher pH value, observed in Figure 7a, loses the isosbestic point; this phenomenon can be attributed to the presence of ionized quinoidal base (A^−^) absorbing at different wavelength than A in equilibrated solutions. 

## 4. Conclusions

SCn exhibit higher association constants with the flavylium cation in simple synthetic compounds than the ones observed for natural anthocyanins. The highest 4′7-DHF *K_AH+SCn_* was obtained with SC4, the host with the smallest cavity, so the cavity size in SC4 seems to be perfect to accommodate this guest. The constant was higher in SC8 than in SC6, but lower when compared with SC4; here, the flexibility and the conformational structure of the hosts seems to be important for complexing the flavylium cation. It was demonstrated that the synthetic guest is hosted into the SCn cavities by the changes observed in the ^1^H NMR chemical shifts of the guest protons located around the C ring. Complexation with the Oenin has a cavity-size dependence: the higher the cavity, the higher the *K_AH+SCn_*_._ The low association constants and/or the anthocyanin aggregation impede significant changes in the NMR ^1^H chemical shifts of the anthocyanin protons after titration with the hosts. The higher selectivity of the SCn hosts for the flavylium cation than for the other species formed at pH > 1 make possible to increase the flavylium domain along the pH scale, with this shift being directly proportional to the *K_AH+SCn_*_._

## Figures and Tables

**Table 1:** Association constants *K_AH+SCn_* (M^−1^) for the formation of 1:1 host-guest complexes between *p*-sulfonated calix[n]arenes and flavylium species of 4′7-DHF and Oenin. *K_AH+SCn_* (M^−1^) calculated after fitting (Equation (1)) of the data obtained after titration of 4′7-DHF (1.2 × 10^−5^ M) and Oenin (2.2 × 10^−5^ M; 1.8 × 10^−5^ M; 1.6 × 10^−5^ M) solutions with increasing concentrations of SC4, SC6 and SC8 at pH = 1.

Compound	SC4	SC6	SC8
4′7-DHF	11400	2200	7700
Oenin	174	224	1100

Exp. Error 20%.

**Table 2:** Association constants *K_CB^SCn_* (M^−1^) for the formation of 1:1 host-guest complexes between *p*-sulfonated calix[n]arenes and the CB^ species of Oenin. *K_CB^SCn_* (M^−1^) calculated after fitting (Equation (2)) of the data obtained after titration of Oenin (2.0 × 10^−5^ M; 1.8 × 10^−5^ M; 1.8 × 10^−5^ M) solutions with increasing concentrations of SC4, SC6 and SC8 at pH ≈ 3.5.

Compound	SC4	SC6	SC8
Oenin	<10	10	67

Exp. Error 20%.

## Data Availability

The data presented in this study are available in supplementary material.

## Supplementary Materials

The following are available online. Figure S1: 1H NMR spectra varia-tions of 4′7OH (6.1 × 10^−3^ M) solution upon titration with increasing concentrations of the host: 0, 0.5, 1.2, 3, 10, and 20 equivalents of SC6. Figure S2: 1H NMR spectra variations of 4′7OH (6.1 × 10^−3^ M) solution upon titration with increasing concentrations of the host: 0, 0.3, 0.6, 1.8, and 13 equivalents of SC8. Figure S3: 1H NMR spectra variations of Oenin (2.2 × 10^−4^ M) solution upon titration with increasing concentrations of the host: 0, 9, 23, 45 and 68 equivalents of SC6. Figure S4: 1H NMR spectra variations of Oenin (2.2 × 10^−4^ M) solution upon titration with increasing concentrations of the host: 0, 9, 23, 45 and 68 equivalents of SC8. Figure S5: UV-Vis spectral variations of equili-brated solutions of 4′7-DHF 1.15 × 10−5 M obtained after direct pH jumps from pH = 1 to higher pH values (citrate buffer 0.01 M). (a) in presence of SC6 64 mM and (b) SC8 28 mM. The insets show the fitting for the calculation of pK’a. Figure S6: UV-Vis spectral variations of pseu-do-equilibrated solutions of Oenin 1.7 × 10−5 M obtained after direct pH jumps from pH = 1 to higher pH values (citrate buffer 0.01M). (a) in presence of SC4 40 mM and (b) SC6 32 mM. The in-sets show the fitting for the calculation of pK^a.

## Sample Availability

Samples of the compounds are not available from the authors.
