# Supplementary material for: Anthocyanin Color Stabilization by Host-Guest Complexation with p-Sulfonatocalix[n]arenes
Source: Molecules. 2021 Sep 4;26(17):5389. doi: 10.3390/molecules26175389 (PMC8434021; doi:10.3390/molecules26175389)
Supplement: Supplementary file 1 [file molecules-26-05389-s001.zip › molecules-1326119-supplementary.pdf]

Anthocyanin Color Stabilization by Host-Guest Complexation with *p*-Sulfonatocalix[n]arenes

Johan Mendoza,[a*] Luis Cruz,[b] Victor de Freitas,[b] Fernando Pina[a] and Nuno Basílio.[a*]

[a] LAQV – REQUIMTE, Departamento de Química, Faculdade de Ciências e Tecnologia, Universidade Nova de Lisboa, 2829-516 Caparica, Portugal, j.chacon@campus.fct.unl.pt; nuno.basilio@fct.unl.pt

[b] LAQV – REQUIMTE, Departamento de Química e Bioquímica, Faculdade de Ciências, Universidade do Porto, Rua do Campo Alegre, 687, 4169-007 Porto, Portugal.

Calculation of the association constants

Equation 1 and 2 were used to calculate the host-guest association constants in complexes with 1:1 stoichiometries, these equations require an excess of the host in comparison with the guest. Following we present the deduction of such equations:

When flavylium cation guest (AH^+) is the sole species in solution (commonly at pH=1) the interaction with the host (SCn) is described as follow:

$$AH^+ + SC_n \leftrightharpoons AH^+SC_n \qquad K_{AH^+SC_n} = \frac{[AH^+SC_n]}{[AH^+][SC_n]} \qquad \text{S1}$$

The mass balance for the species containing the guest.

$$C_0 = [AH^+] + [AH^+SC_n] = [AH^+](1 + K_{AH^+SC_n}[SC_n]) \qquad \text{S2}$$

The molar fraction of the species is calculated:

$$\chi_{AH^+} = \frac{[AH^+]}{C_0} = \frac{1}{1+K_{AH^+SC_n}[SC_n]} \qquad \text{S3}$$

$$\chi_{AH^+SC_n} = \frac{[AH^+SC_n]}{C_0} = \frac{K_{AH^+SC_n}[SC_n]}{1+K_{AH^+SC_n}[SC_n]} \qquad \text{S4}$$

As the absorbance follows the Lambert-Beer law, we can express it as follow:

$$A_\lambda = C_0(\varepsilon_{AH^+}\chi_{AH^+} + \varepsilon_{AH^+SC_n}\chi_{AH^+SC_n}) \qquad \text{S5}$$

And then, introducing the parameter $r_{AH^+SC_n}$:

$$r_{AH^+SC_n} = \frac{\varepsilon_{AH^+SC_n}}{\varepsilon_{AH^+}} \qquad \text{S6}$$

Being ε_{AH^+} and $\varepsilon_{AH^+SC_n}$ the molar absorption coefficient of the free flavylium and the complexed respectively. Including the mole fraction in Eq. S5, we have equation 1, presented in the main text, where the parameters $r_{AH^+SC_n}$ and $K_{AH^+SC_n}$ are fitted with the experimental data.

On the other hand, Equation 2 is deduced taking in account the possible interaction of the conjugate base (CB) species with the macrocycle.

$$AH^+ + H_2O \leftrightharpoons CB + H_3O^+ \qquad K'_a = \frac{[CB][H^+]}{[AH^+]} \qquad \text{S7}$$

$$CB + SC_n \leftrightharpoons CBSC_n \qquad K_{CBSC_n} = \frac{[CBSC_n]}{[CB][SC_n]} \qquad \text{S8}$$

The mass balance would be:

$$\begin{aligned} C_0 &= [AH^+] + [AH^+SC_n] + [CB] + [CBSC_n] \\ &= [AH^+](1 + K_{AH^+SC_n}[SC_n] + \frac{(1+K_{CBSC_n}[SC_n]K'_a}{[H^+]}) \end{aligned} \qquad \text{S9}$$

And the mole fraction calculated as:

$$\chi_{AH^+} = \frac{[H^+]}{\left(1+K_{AH^+SC_n}[SC_n]\right)[H^+]+(1+K_{CBSC_n}[SC_n])K'_a} = \frac{[H^+]}{D} \qquad \text{S10}$$

$$\chi_{AH^+SC_n} = \frac{K_{AH^+SC_n}[SC_n][H^+]}{D} \qquad \text{S11}$$

$$\chi_{CB} = \frac{K'_a}{D} \qquad \text{S12}$$

$$\chi_{CBSC_n} = \frac{K_{CBSC_n}K'_a[SC_n]}{D} \qquad \text{S13}$$

The Lambert-Beer law, can be expressed as:

$$A_\lambda = C_0(\varepsilon_{AH^+}\chi_{AH^+} + \varepsilon_{AH^+SC_n}\chi_{AH^+SC_n} + \varepsilon_{CB}\chi_{CB} + \varepsilon_{CBSC_n}\chi_{CBSC_n}) \qquad \text{S14}$$

And then, introducing the parameters r_{CB} and r_{CBSC_n}:

$$r_{CB} = \frac{\varepsilon_{CB}}{\varepsilon_{AH^+}} \qquad r_{CBSC_n} = \frac{\varepsilon_{CBSC_n}}{\varepsilon_{AH^+}} \qquad \text{S15}$$

Being ε_{CB} and ε_{CBSC_n} the molar absorption coefficient of the free CB species and the complexed respectively. Including the mole fraction in Eq. S14, we have equation 2, where the parameters r_{CBSC_n} and K_{CBSC_n} are fitted with the experimental data, while the r_{CB} can be obtained from the initial absorbance when $[SC_n]$=0.

$$A_{\lambda(SC_n=0)} = A_0(\frac{[H^+]+r_{CB}}{[H^+]+K'_a}) \qquad \text{S16}$$

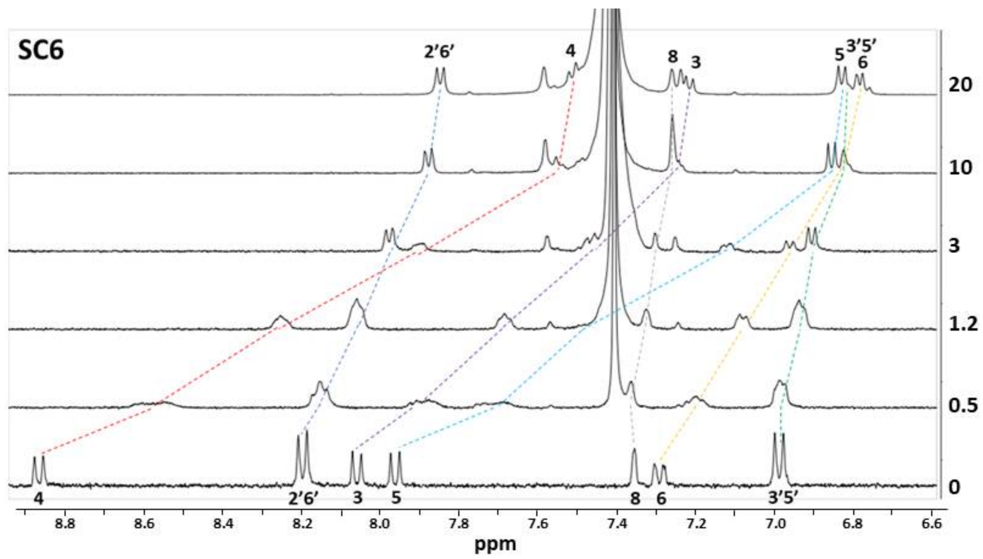

Figure S1. ^{1}H NMR spectra variations of 4'7OH (6.1×10^{-3} M) solution upon titration with increasing concentrations of the host: 0, 0.5, 1.2, 3, 10, and 20 equivalents of SC6.

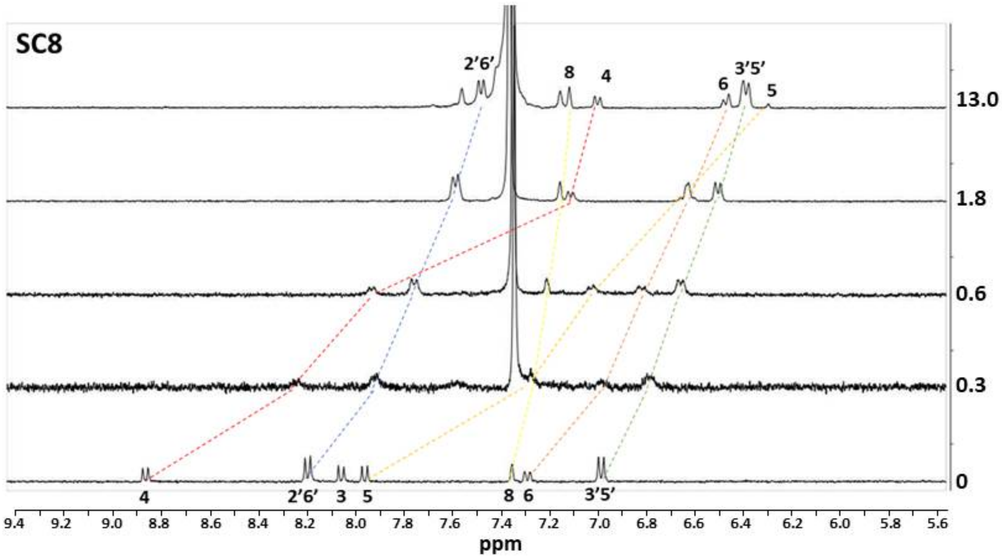

Figure S2. ^{1}H NMR spectra variations of 4'7OH (6.1×10^{-3} M) solution upon titration with increasing concentrations of the host: 0, 0.3, 0.6, 1.8, and 13 equivalents of SC8.

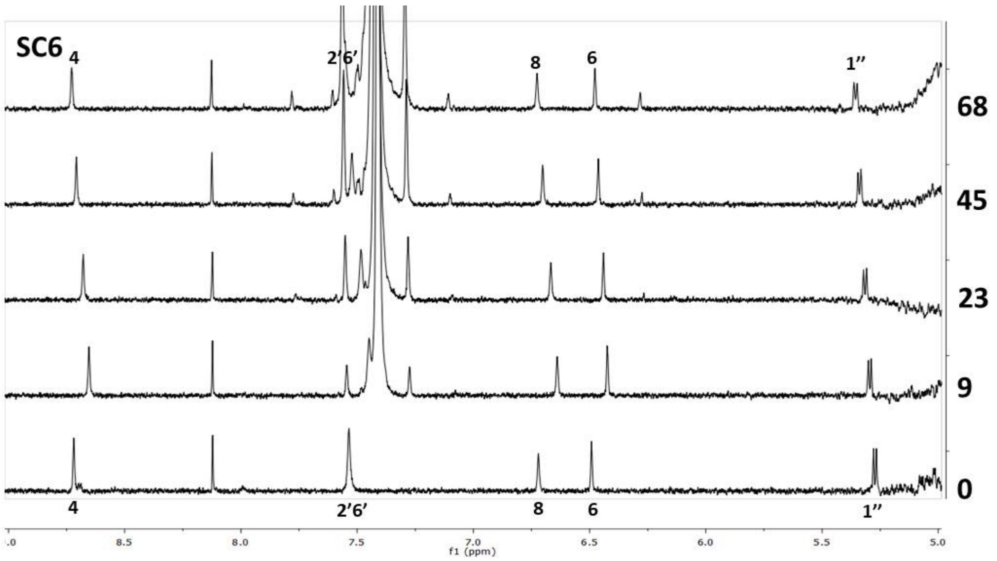

Figure S3. ^{1}H NMR spectra variations of Oenin (2.2×10^{-4} M) solution upon titration with increasing concentrations of the host: 0, 9, 23, 45 and 68 equivalents of SC6.

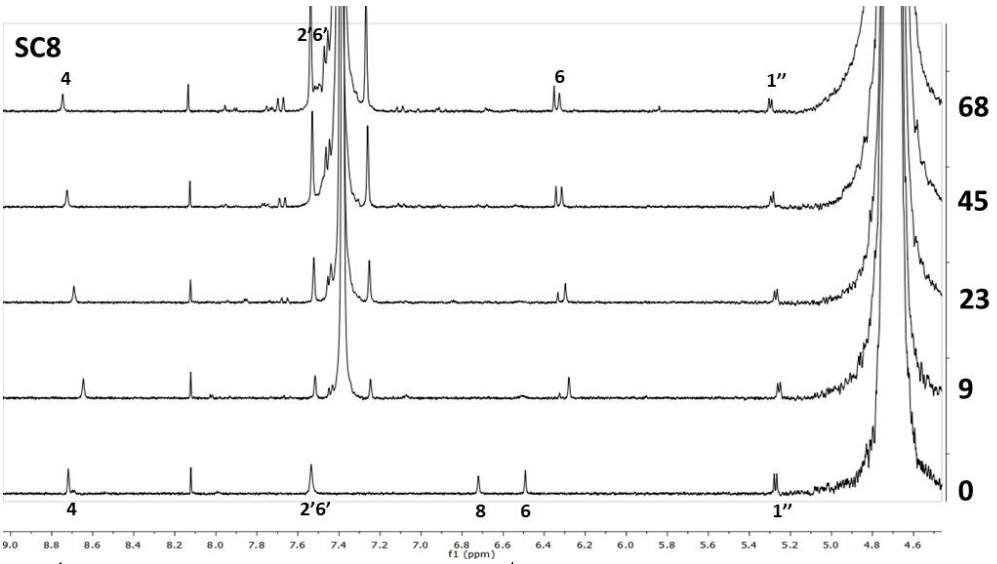

Figure S4. ^{1}H NMR spectra variations of Oenin (2.2×10^{-4} M) solution upon titration with increasing concentrations of the host: 0, 9, 23, 45 and 68 equivalents of SC8.

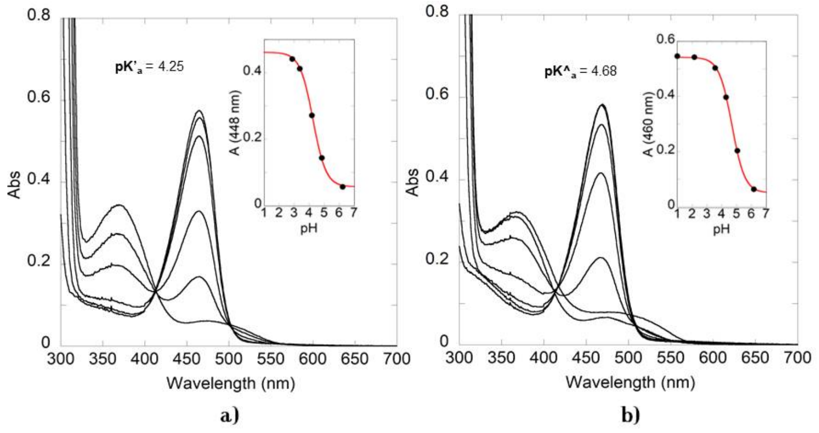

Figure S5. UV-Vis spectral variations of equilibrated solutions of 4'7-DHF 1.15×10^{-5} M obtained after direct pH jumps from pH=1 to higher pH values (citrate buffer 0.01M). a) in presence of SC6 64 mM and b) SC8 28 mM. The insets show the fitting for the calculation of pK'_a.

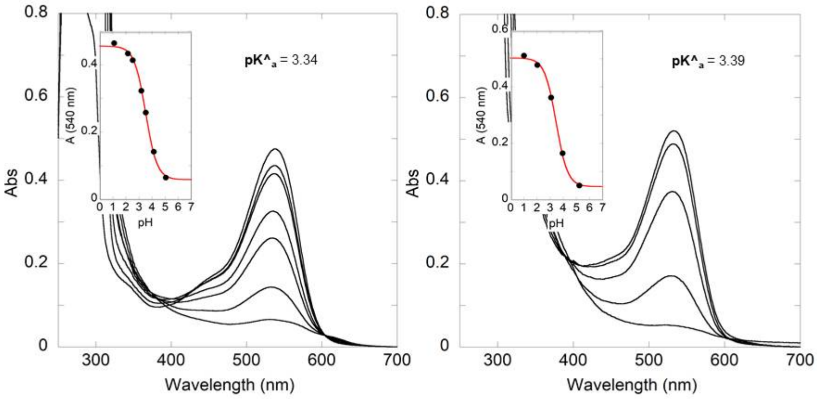

a) **b)**

Figure S6. UV-Vis spectral variations of pseudo-equilibrated solutions of Oenin 1.7×10^{-5} M obtained after direct pH jumps from pH=1 to higher pH values (citrate buffer 0.01M). a) in presence of SC4 40 mM and b) SC6 32 mM. The insets show the fitting for the calculation of p$K^{\wedge}_{a}$.
